# Prevalence and Genetic Characterization of *Cryptosporidium, Giardia* and *Enterocytozoon* in Chickens From Ezhou, Hubei, China

**DOI:** 10.3389/fvets.2020.00030

**Published:** 2020-01-31

**Authors:** Shengkui Cao, Meng Xu, Yanyan Jiang, Hua Liu, Zhongying Yuan, Lei Sun, Jianping Cao, Yujuan Shen

**Affiliations:** ^1^National Institute of Parasitic Diseases, Chinese Center for Disease Control and Prevention, Shanghai, China; ^2^Chinese Center for Tropical Diseases Research, Shanghai, China; ^3^Key Laboratory of Parasite and Vector Biology, Ministry of Health, Shanghai, China; ^4^WHO Collaborating Centre for Tropical Diseases, Shanghai, China; ^5^National Center for International Research on Tropical Diseases, Ministry of Science and Technology, Shanghai, China

**Keywords:** *Cryptosporidium*, *Giardia intestinalis*, *Enterocytozoon bieneusi*, assemblage C, genotype D

## Abstract

*Cryptosporidium* spp., *Giardia* spp. and microsporidia are important intestinal protozoa responsible for diarrhea in humans and other mammals. China is a major chicken-raising country, and studies on these protozoa in chickens have important public health significance. Here, we investigated the prevalence and genetic characterization of these parasites in chickens from Ezhou City, Hubei Province, China. In total, 206 stool specimens were collected from chickens in four villages of Ezhou between July 2014 and February 2015. Genomic DNA of each specimen was tested by nested PCR based on the *Cryptosporidium* small subunit rRNA gene, the *Giardia intestinalis* triose phosphate isomerase gene, and the internal transcribed spacer of the *Enterocytozoon bieneusi* rRNA gene, respectively. The public health significance of *G. intestinalis* and *E. bieneusi* identified in our study was evaluated via phylogenetic analysis. The infection rates were determined to be 2.43% (5/206), 8.25% (17/206), and 1.94% (4/206) for *Cryptosporidium, G. intestinalis*, and *E. bieneusi*, respectively. One sample showed coinfection with *G. intestinalis* and *E. bieneusi*. Meanwhile, sequence analysis of the PCR-positive samples showed that the *Cryptosporidium* was *C. baileyi, G. intestinalis* was assemblage C, and *E. bieneusi* was genotype D and novel genotype EZ0008. This is the first report of zoonotic *G. intestinalis* assemblage C in chickens in the world, and the first report of zoonotic *E. bieneusi* genotype D in chickens in China. These findings indicate new transmission dynamics and molecular epizootiology.

## Introduction

*Cryptosporidium* spp., *Giardia* spp. and microsporidia are important intestinal protozoa of humans, livestock, and wild animals, which cause acute or self-limiting diarrhea (1–3). The diseases caused by infection with these parasites are distributed worldwide (4, 5). *Cryptosporidium* spp. often infect immunodeficient patients (especially HIV-infected persons), children and the elderly, which can be fatal (6, 7). *Giardia intestinalis* (*G. intestinalis*), which is the etiologic agent of giardiasis, usually appears in tourists, resulting in diarrhea called “Traveler's Diarrhea.” Zoonotic giardiasis is one of the ten principal parasitoses threatening human health worldwide (1). Microsporidia is mainly associated with immunocompromised individuals, causing wasting syndrome. One of the most frequently identified microsporidian species in fecal samples of clinical patients and domestic animals worldwide is *Enterocytozoon bieneusi* (*E. bieneusi*) (8). Many outbreaks among humans have been caused by these parasites (9–13), for example, the massive *Cryptosporidium*-associated waterborne outbreak in Milwaukee, Wisconsin in 1993 (14). Such outbreaks pose significant challenges to public health. In the United States, it is estimated that 748,000 cryptosporidiosis cases occur every year (15). According to the World Health Organization, annually, there are 500,000 emerging giardiasis cases globally. The prevalence of human microsporidiosis ranges from 0 to 50% depending on the geographical region (16).

Epidemiological data on human cryptosporidiosis, giardiasis and microsporidiosis has confirmed their common occurrence in China (6, 17). For example, the occurrence rates of these three parasitoses were 13.49, 6.75, and 13.49% respectively in outpatients suffering from diarrhea in Shanghai, China in 2014 (6) and 2.0, 1.4, and 0.2% respectively in children with a history of diarrhea in Hubei province, China in 2017 (13).

Poultry play a significant role in the agricultural economy of China. As a major chicken-raising country, the total population of layer chicken was 2.6 billion in 2013, accounting for 37.3% of the total number in the world (http://kids.fao.org/glipha/). Up to now, there is limited information regarding the distribution and molecular characterization of *Cryptosporidium* spp., *G. intestinalis*, and *E. bieneusi* in chickens in China. Due to transmission by these parasites is the fecal-oral route, often through direct contact with feces from infected animals or people, contaminated food and/or water (18), together with the close contact between chickens and humans in rural areas, knowledge of parasitic species in chickens has important public health significance.

In the present study, we examined the occurrence of *Cryptosporidium* spp., *G. intestinalis* and *E. bieneusi* in chickens from four villages in Ezhou City, Hubei Province, China, identified the species/genotypes of these intestinal protozoa, and assessed their potential for zoonotic transmission. Furthermore, the public health significance of *G. intestinalis* and *E. bieneusi* identified in our study was evaluated via phylogenetic analysis.

## Materials and Methods

### Specimen Collection

Between July 2014 and February 2015, 206 fresh stool specimens were collected from chickens on four different farms from villages in Ezhou City, Hubei Province, China. Animal details, including their location, age and sampling time, were recorded. The chickens ranged in age from 2 months to 1 year old. On these farms, they were kept separately in individual cages, and fresh fecal excretion was collected from cages with care, avoiding contamination from other cages. Each sample was >5 g.

All specimens were taken to laboratory in a cool box at 4°C, registered and stored at −20°C until DNA extraction.

### DNA Extraction

Sufficient specimens (200–300 mg of each stool specimen) were used for DNA extraction and purification using a QIAamp® Fast DNA Stool Mini Kit (QIAGEN, Hilden, Germany), following the manufacturer-recommended procedures. The extracted genomic DNA samples were stored at −20°C before polymerase chain reaction (PCR).

### Parasite Identification in Animal Samples

*Cryptosporidium* spp., *G. intestinalis*, and *E. bieneusi* in the fecal specimens were detected using individual nested PCR and the sequences analyzed were of the small subunit (SSU) rRNA gene, the triose phosphate isomerase (*tpi*) gene, and the internal transcribed spacer (ITS) of the rRNA gene, respectively. The primers for each parasite referred to previous descriptions (6, 19–21).

The PCR was conducted in a 25 μl reaction mixture including 12.5 μl *Taq* mix (2×), 11.3 μl nuclease-free water, 1 μl genomic DNA template (20–60 ng/μl), 0.1 μl of sense and antisense primers each (100 μM). For the nested PCR, 1 μl of the first PCR product was used as the template. The genes from *Cryptosporidium* spp. and *G. intestinalis* were amplified using GoTaq® Green Master Mix (Promega, code no. M7123, Madison, WI, USA), while the gene from *E. bieneusi* was amplified using Premix *Taq*® (Takara, code no. RR901M, Dalian, China).

The PCR cycling condition for *Cryptosporidium* spp. was as follows: denaturation at 94°C for 1 min, followed by 35 cycles (94°C for 10 s, 55°C for 30 s and 72°C for 1 min) and a final extension step at 72°C for 10 min. The secondary reaction was carried out similarly. The cycling condition for *G. intestinalis* was: denaturation at 94°C for 5 min, followed by 35 cycles (94°C for 45 s, 57.5°C for 45 s, and 72°C for 1 min) and a final extension step at 72°C for 7 min. For *E. bieneusi*, the cycling condition was the same to *G. intestinalis*, except the annealing temperature was 55°C. The secondary reactions were identical to the primary PCR cycling conditions.

Each DNA sample was analyzed three times to ensure the reliability of results with positive (templates were positive nucleic acids of *Cryptosporidium, G. intestinalis* and *E. bieneusi* stored in our laboratory) and negative (template was nuclease-free water) controls in each PCR. All final secondary PCR products were visualized by electrophoresis in 2% agarose gels after ethidium bromide staining.

### Sequencing of Positive Genes

Positive PCR products were treated by a Big Dye Terminator v3.1 Cycle Sequencing kit (Applied Biosystems, Foster City, USA), and sequenced in both directions on an ABI 3730 DNA analyzer (Applied Biosystems), using the secondary primers. Sequencing was performed by the Shanghai Sunny Biotechnology Co., Ltd. (Shanghai, China).

### DNA Sequences and Statistical Analysis

ContigExpress was used to evaluate the wave peak and assemble the sequences. Each sequence was compared against sequences in the NCBI database and analyzed using Clustal X 1.83 and MEGA 5, determining the species/genotypes of parasites. To assess the phylogenetic relationships of the sequences identified in our study and the known ones, the neighbor-joining analyses of *G. intestinalis* assemblages at the *tpi* locus and *E. bieneusi* genotypes at the ITS locus were calculated by the Kimura two-parameter model, and 1,000 replicates were used. All statistical analyses were performed using SPSS version 17.0 (SPSS Inc., Chicago, IL). Association of age category and parasitic infections was analyzed using the chi-square test, and *P* < 0.05 was considered statistically significant.

## Results

### Occurrence of *Cryptosporidium* spp., *G. intestinalis*, and *E. bieneusi*

In chickens (*n* = 206), the positive rates of *Cryptosporidium, G. intestinalis*, and *E. bieneusi* determined using nested PCR were 2.43% (5/206), 8.25% (17/206), and 1.94% (4/206), respectively (Table 1). Polyparasitism was observed in one specimen from village A, which was coinfected with *G. intestinalis* and *E. bieneusi*. Specimens positive for *Cryptosporidium* and *E. bieneusi* were not restricted to a particular village, whereas *G. intestinalis* was only detected in village A (Table 2). No obvious age-associated difference in parasitic infections was observed in the chickens (*P* > 0.05).

**Table 1:** Occurrence of *Cryptosporidium, Giardia*, and microsporidia.

**Genus**	**Number of positive specimens (*n*%)**	**Species**	**Genotype**
*Cryptosporidium*	5 (2.43%)	*C. baileyi*	–
*Giardia*	17 (8.25%)	*G. intestinalis*	Assemblage C
*Enterocytozoon*	4 (1.94%)	*E. bieneusi*	Genotype D (*n* = 2)/EZ0008 (*n* = 2)
Total specimens	206		

**Table 2:** Distribution of positive samples in different villages.

**Village**	**Number of specimens**	***Cryptosporidium***	***G. intestinalis***	***E. bieneusi***
A	151	–	17 (11.3%)	1 (0.66%)
B	20	4 (20.0%)	–	–
C	19	–	–	2 (10.5%)
D	16	1 (6.25%)	–	1 (6.25%)

*Samples were analyzed by nested PCR*.

### Molecular Analyses of the Parasites

Five *Cryptosporidium*-positive specimens were identified by nested PCR. DNA sequencing followed by alignment of the SSU rRNA gene fragments revealed these isolates belonged to *C. baileyi*. Of them, two sequences (four cases: KY448454, KY448455, KY448457, and KY448458) showed 100% homology with that previously reported, and one novel sequence (KY448456) was found.

Seventeen specimens were identified as *G. intestinalis*-positive by nested PCR. Sequence analysis of the *tpi* gene indicated that these isolates belonged to assemblage C. Among them, five sequences (13 cases: KY448449, KY448450, KY448460-KY448469, and KY448471) were 100% identical to that previously reported, and two novel sequences (four cases: KY448459/KY448448 and KY448447/KY448470) were found.

DNA sequencing and analysis of the ITS gene by nested PCR showed that the two *E. bieneusi*-positive specimens (KY448446 and KY448451) were identical to zoonotic genotype D. One novel genotype (two cases: KY448452 and KY448453) was found and named EZ0008.

### Phylogenetic Analysis

**Figure 1:**
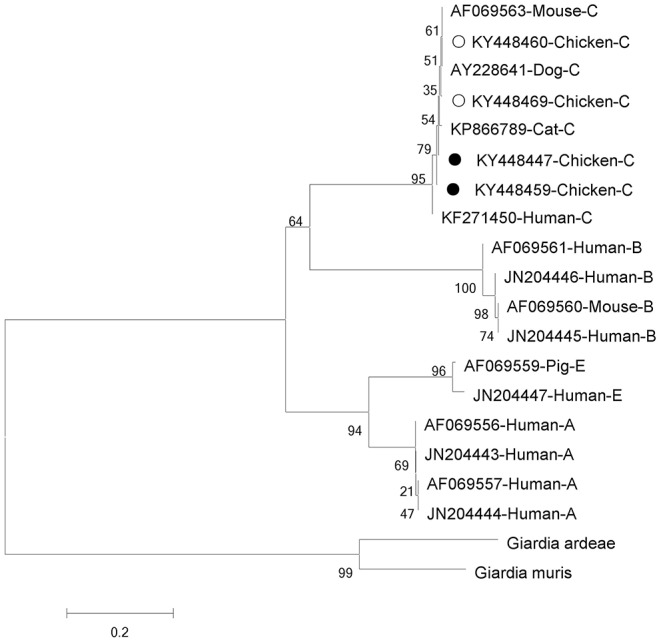
Phylogenetic relationships between assemblages of *Giardia intestinalis*. The relationships were inferred using neighbor-joining analysis of triose phosphate isomerase (*tpi*) sequences based on genetic distance calculated by the Kimura two-parameter model. Each sequence is marked with its accession number, host origin and assemblage. The numbers on the branches are percentage bootstrapping values from 1,000 replicates. Open and solid circles, respectively, indicate previously known and novel sequences identified in this study.

**Figure 2:**
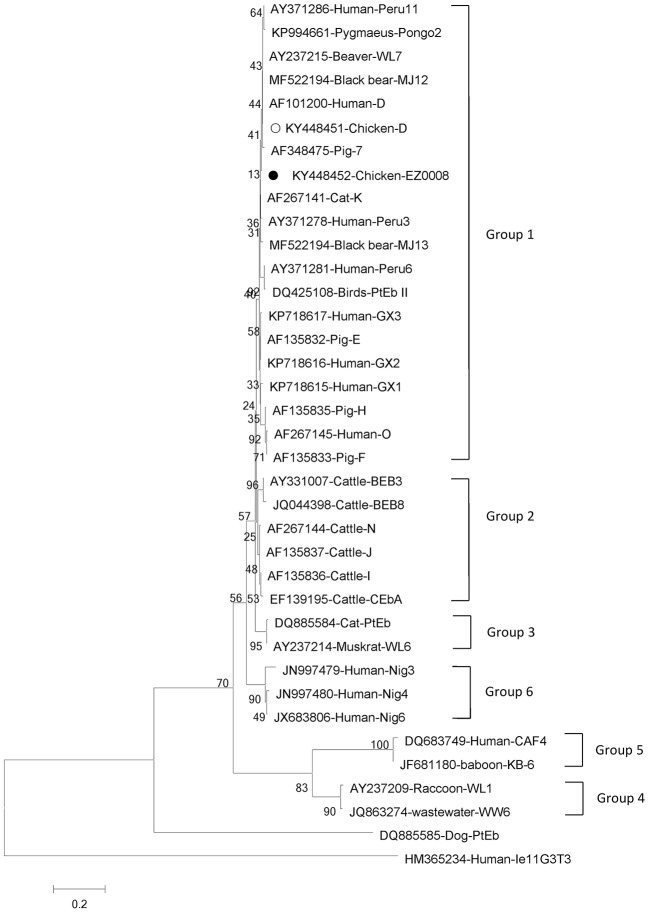
Phylogenetic relationships between genotype groups of *Enterocytozoon bieneusi*. The relationships were inferred using neighbor-joining analysis of internal transcribed spacer (ITS) sequences based on genetic distance calculated by the Kimura two-parameter model. Each sequence is marked with its accession number, host origin and genotype designation. The numbers on the branches are percentage bootstrapping values from 1,000 replicates. Open and solid circles, respectively, indicate previously known and novel sequences identified in this study.

Phylogenetic analyses of *G. intestinalis* and *E. bieneusi* were performed to understand the relationships on the basis of published nucleotide sequences. The two novel sequences of *G. intestinalis* belong to assemblage C, which can infect mice, dogs, cats, and humans (Figure 1). The novel genotype of *E. bieneusi* was phylogenetically related to Group 1, which includes most of the human pathogenic genotypes (Figure 2).

## Discussion

To date, 38 species of *Cryptosporidium* have been identified in diverse hosts, totaling more than 40 subtypes infecting mammals (22–24). Among these, *C. hominis* and *C. parvum* are the main pathogens infecting humans and responsible for approximately 90% of human cryptosporidiosis (25). The reported main species of *Cryptosporidium* in chickens include *C. meleagridis, C. baileyi* (26, 27), *C*. *parvum* (28), *C. galli* (29), and avian genotype II (30), and *C. meleagridis, C*. *parvum* are zoonotic. In this study, only *C. baileyi* was identified in chickens, and the occurrence rate was 2.43%, which was in accord with previous reports (0–33.3% in China, 0.7% in Iran, 4.8% in Jordan) (27, 31, 32). One novel sequence (KY448456) was found, which has a high homology with isolate GU377273 from ostrich, based on sequence alignment.

Based on genetic analysis, *G. intestinalis* has been grouped into 8 assemblages (A–H) (33). Assemblages A and B are the major genotypes infecting humans, and assemblages C (6), E (34), and F (35) also infect people. Only zoonotic genotypes of assemblages A and B have been reported in chickens (36). Interestingly, all *G. intestinalis*-positive chickens on farm A in this study were found to be infected with assemblage C. Assemblage C of *G. intestinalis* usually occurs in dogs (4, 37), and occasionally in cats (38), coyotes (39), and humans (6). To our knowledge, this is the first report of assemblage C in chickens. Two novel sequences (four cases: KY448459/KY448448 and KY448447/KY448470) were identified, which, based on sequence alignment and phylogenetic analysis, have high homology with isolate KP866789 from cat, and also have homology with isolate KF271450 from human, indicating public health significance. A survey of diarrhea patients in Shanghai, China, in 2013 indicated that the infection rate with assemblage C was up to 6.35% (6). Thus, *G. intestinalis*-infected chickens might pose a great risk of human infection in this area. More specimens and deeper study should be undertaken to understand the transmission dynamics.

*E. bieneusi* has about 11 genotype groups, and Group 1 contains most zoonotic genotypes; the other groups contain mostly host-adapted genotypes (40). Several genotypes of *E. bieneusi* in chickens have been reported, including genotype J in Germany (41), genotype Peru8 in Brazil (42), genotypes Henan-IV, and CC-1 in China (43) and genotypes Peru6, Peru11, Type IV, and D in Brazil (44), which are zoonotic except for CC-1. *E. bieneusi* genotype D belongs to Group 1 and is the most common zoonotic genotype, which has been found in humans, dogs, cats, rhesus monkeys, some livestock and wild mammals (18, 21). The genotype is also widespread in wastewater (45). The first report of genotype D infecting chickens came from Minas Gerais, Brazil (9.27%) (44). However, only one report has been published concerning *E. bieneusi* infection in chickens in China, with genotypes Henan-IV and CC-1 (43). In our study, genotype D and novel EZ0008 were identified for the first time in chickens in China, suggesting multi-genotype infections of *E. bieneusi* in chickens in this country. The novel genotype (two cases: KY448452 and KY448453) was related to Group 1 and has high homology with isolate AF348475 from pig and isolate AF101200 from human, indicating public health significance. Our results imply that humans in rural areas are at significant risk of infection by *E. bieneusi* because of intimate contact with livestock.

Overall, chickens from Ezhou were determined to carry *Cryptosporidium, G. intestinalis*, and *E. bieneusi*. Among the parasites detected, species of *G. intestinalis* assemblage C and *E. bieneusi* genotype D are zoonotic, and can be transmitted through water or food, resulting in giardiasis and microsporidiosis, respectively. Presently, prevention is the predominant measure to control these diseases. Knowledge of the parasite genetic profile, source of infection, mode of transmission and susceptible population is beneficial for effective control. Considering these zoonoses in Ezhou, Hubei, China, peasants should prevent direct/indirect contact with chicken feces, and also avoid discharging these feces to nearby water, because contamination of water sources is the principal cause of outbreaks and prevalence of these parasitic diseases (46). Zoonotic species of *G. intestinalis* and *E. bieneusi*/EZ0008 were identified in villages A, C, and D in this study (Table 2); in particular, we found numerous examples of *G. intestinalis* assemblage C in village A. Although no zoonotic parasites were detected in village B in our study, their presence cannot be excluded because the sample number was small. In future, we will investigate the occurrence rates of these three parasites in humans (especially diarrhea patients) and waters in Ezhou, to clarify their patterns of transmission and help with risk control. In summary, the present study provides important reference data on the epidemiology of *Cryptosporidium, G. intestinalis*, and *E. bieneusi* infections in chickens.

## Data Availability Statement

The nucleotide sequences generated in present study were submitted to the NCBI GenBank with accession numbers KY448446 to KY448471.

## Ethics Statement

Ethical clearance for the collection and examination of chicken feces samples was obtained from the Guide for the Care and Use of Laboratory Animals of the National Institute of Parasitic Diseases, Chinese Center for Disease Control and Prevention. The protocol was approved by the Laboratory Animal Welfare & Ethics Committee (LAWEC), National Institute of Parasitic Diseases, Chinese Center for Disease Control and Prevention, China (reference no. 2012-12). Before beginning our work, we contacted the farm owners and obtained their permission. No specific permits were required for the described field studies. We directly collected fecal specimens from the cages using plastic bags, requiring very little contact with the chickens. The chickens were not harmed in any way during the procedure.

## Author Contributions

YS, SC, and JC conceived and designed the experiments. SC, YJ, HL, ZY, and LS performed the experiments. MX and SC analyzed the data. SC wrote the manuscript. YS and JC revised the manuscript. All authors read and approved the final version of the manuscript.

### Conflict of Interest

The authors declare that the research was conducted in the absence of any commercial or financial relationships that could be construed as a potential conflict of interest.
